# A new tool for assessing Pectus Excavatum by a semi-automatic image processing pipeline calculating the classical severity indexes and a new marker: the Volumetric Correction Index

**DOI:** 10.1186/s12880-022-00754-0

**Published:** 2022-02-20

**Authors:** Rosella Trò, Simona Martini, Nicola Stagnaro, Virginia Sambuceti, Michele Torre, Marco Massimo Fato

**Affiliations:** 1 Department of Informatics, Bioengineering Robotics and System Engineering (DIBRIS), University of Genoa, Viale Causa 13, 16143 Genova, Italy; 2 Complex Operative Radiology Unit, IRCCS Giannina Gaslini, Genova, Italy; 3 Department of Health Sciences (DISSAL), Radiodiagnostics, University of Genoa, Via A. Pastore 1, 16132 Genova, Italy; 4 Pediatric Thoracic and Airway Surgery Unit, IRCCS Giannina Gaslini, Genova, Italy

**Keywords:** Pectus Excavatum, Magnetic resonance imaging, Image processing pipeline

## Abstract

**Background:**

In clinical assessment of Pectus Excavatum (PE), the indication to surgery is based not only on symptoms but also on quantitative markers calculated from Computed Tomography (CT) or Magnetic Resonance Imaging (MRI) scans. According to clinical routine, these indexes are measured manually by radiologists with limited computer support. This process is time consuming and potentially subjected to inaccuracy and individual variability in measurements. Moreover, the existing indexes have limitations, since they are based on linear measurements performed on single slices rather than on volumetric data derived from all the thoracic scans.

**Results:**

In this paper we present an image processing pipeline aimed at providing radiologists with a computer-aid tool in support of diagnosis of PE patients developed in MATLAB® and conceived for MRI images. This framework has a dual purpose: (i) to automatize computation of clinical indexes with a view to ease and standardize pre-operative evaluation; (ii) to propose a new marker of pathological severity based on volumetric analysis and overcoming the limitations of existing axial slice-based indexes. Final designed framework is semi-automatic, requiring some user interventions at crucial steps: this is realized through a Graphical User Interface (GUI) that simplifies the interaction between the user and the tools. We tested our pipeline on 50 pediatric patients from Gaslini Children’s Hospital and performed manual computation of indexes, comparing the results between the proposed tool and gold-standard clinical practice. Automatic indexes provided by our algorithm have shown good agreement with manual measurements by two independent readers. Moreover, the new proposed Volumetric Correction Index (VCI) has exhibited good correlation with standardized markers of pathological severity, proving to be a potential innovative tool for diagnosis, treatment, and follow-up.

**Conclusions:**

Our pipeline represents an innovative image processing in PE evaluation, based on MRI images (radiation-free) and providing the clinician with a quick and accurate tool for automatically calculating the classical PE severity indexes and a new more comprehensive marker: the Volumetric Correction Index.

**Supplementary Information:**

The online version contains supplementary material available at 10.1186/s12880-022-00754-0.

## Background

Pectus Excavatum (PE) is the most common congenital chest-wall deformity in children [1]. It is characterized by a sunken deformity of the anterior chest wall, involving both sternum and costal cartilages. The deformity worsens during adolescence and is primarily male-dominated, with a male/female ratio of 5:1 [2]. Although originally considered an aesthetic condition without clinical implications, several studies conducted in the past decades have demonstrated that PE has a substantial psychosocial impact among developing children [3] and may also lead to disabling cardiopulmonary manifestations in worst cases [4–6]. Indeed, when the deformity is moderate to severe, it can reduce the volume of the chest, restrict the pulmonary movement, and force the heart into a rotated position [7]. These important cardiopulmonary implications can be substantially improved with surgical correction [5, 6, 8, 9].

In order to assess the severity of the malformation and determine treatment options, including surgical repair, patients with PE are evaluated through thoracic imaging, particularly Computed Tomography (CT) and Magnetic Resonance Imaging (MRI). These imaging modalities allow to extract several indexes used as markers to quantify the degree of severity [10]. For years, CT has been the gold standard for preoperative evaluation of PE, providing bone details, anatomic relations, and an option for 3D reconstruction [11–16]. However, considering the young age of PE patients, the efforts to avoid unnecessary radiation exposure should be maximized [11, 17]. Additionally, CT provides static results, which do not allow to know the changes in chest compression during the breathing cycle. A dynamic measurement during the normal respiratory cycle is only possible with a high radiation dose, that should be avoided in such young patients [18, 19].

For these reasons, in the last decade MRI has acquired an important role in the assessment of this pathology. Several studies have validated this modality as an alternative radiation-free diagnostic tool for the assessment of malformation indexes [19–23].

Despite the variety of MRI sequences adopted, all aforementioned works agreed to prove reliability, feasibility, and image quality of fast chest MRI protocols for preoperative evaluation of PE. Indeed, they showed that severity indexes of chest deformity collected from CT scan and fast MRI were comparable. They also highlighted the ability of chest MRI to detail anatomical information such as displacement and rotation of the heart or great vessels anomalies, promoting the adoption of this modality in pre operative workup for patients with PE.

A particular MRI technique, the Cardiac Magnetic Resonance Imaging (CMRI) [24], represents an added value in the evaluation of the influence of sternum impingement on cardiac function [25, 26]. Specifically, CMRI allows for a careful surgical evaluation and preoperative cardiac function assessment, overcoming technical difficulty as well as subjectivity inherent to cardiac ultrasound imaging [27]. Despite being the gold standard to evaluate the cardiac function for all cardiopathies, the use of CMRI in patients with PE dates to recent times.

The first study which propelled momentum for CMRI to be used in preoperative assessment of PE dates to 2010. Saleh et al. [28] showed how CMRI could unravel findings associated with severe PE condition not detectable with cardiac ultrasound, corresponding to a significant reduction of the Right Ventricular Ejection Fraction (RVEF) along with a distortion in the right ventricle geometry. Similar findings were confirmed in a more recent study by Dore et al. [18].

In 2013, Humphries et al. [29] employed CMRI for perioperative evaluation of sternal eversion technique used for PE repair. They found improvement of anatomical chest wall contour and cardiac function, suggesting once again CMRI as a promising tool for delineating the anatomical and physiological components of PE as well as measuring the results of surgical repair.

More recently, Deviggiano et al. [25] combined CT and CMRI modalities to evaluate the impact of the malformation severity on both morphological and functional cardiac parameters, respectively. Patients affected by PE showed significant alterations of cardiac morphology and function that were related to the severity of the deformation and that manifested as an exaggerated interventricular dependence.

In 2019, Vina et al. [26] demonstrated an excellent agreement between chest CT and standard CMR for the evaluatiom of chest wall malformations, thus potentially enabling preoperative assessment of PE severity and cardiac involvement with a single non-invasive diagnostic tool.

In the same year, Lai et al. [30] showed that, in patients with mild PE deformity and minimal symptoms at rest, cardiac MRI might reveal additional functional information than echocardiography, able to explain exertional symptoms. They also demonstrated resolution of cardiac dysfunction with surgical repair of PE.

In 2021, Stagnaro et al. [31] analyzed cardiovascular effects beside of thoracic indexes with multiparametric CMR, using a simple noninvasive device mimicking the immediate, temporary effect of surgical correction with the Vacuum Bell (VB).

If some attempts to automatize image processing of CT scans of PE patients have been made very recently [15, 32], in all existing MRI studies, to the best of or knowledge, the chest-wall malformation indices are manually computed by radiologists. According to a commonly adopted standard procedure, the latter measure specific thoracic distances with a ruler on axial images of the patient’s chest on a standard DICOM viewer for medical images. These thoracic measures are then used to calculate clinical indexes according to their specific formula [33]]. The critical points of this working method are long processing time, low reliability and low reproducibility in measurements [14].

The aim of our work is to develop an image processing framework for evaluation of PE using Magnetic Resonance Imaging (MRI), which can support, standardize and accelerate the diagnostic assessment of patients. Firstly, we want to automatize the computation of existing indexes on MRI images, given the lack of automatic procedures for MRI modality. The other purpose is to introduce an innovative marker of pathological severity, based on a volumetric analysis to quantify chest depression. Indeed, most of the existing clinical indexes are calculated on a single slice, usually corresponding to maximum sternal depression [26]. Thus, accuracy of these indexes largely depends on which images are chosen and how measurements are performed from them. This fact could determine a high degree of variability of measured indexes. Specifically, we want to elaborate an image processing method that first corrects the depression, by simulating the normal morphology of the chest, and then obtains the amount of depression by comparing the images of thorax before and after the image correction. The ratio between the depression volume and the chest volume post-correction gives the portion of chest that must be repaired. This new measure, that we named Volumetric Correction Index (VCI), could represent a more comprehensive marker, complementary to existing clinical indices, of effective patient pre-treatment condition, assisting physicians in diagnosis process and proper treatment choice.

## Implementation

**Fig. 1:**
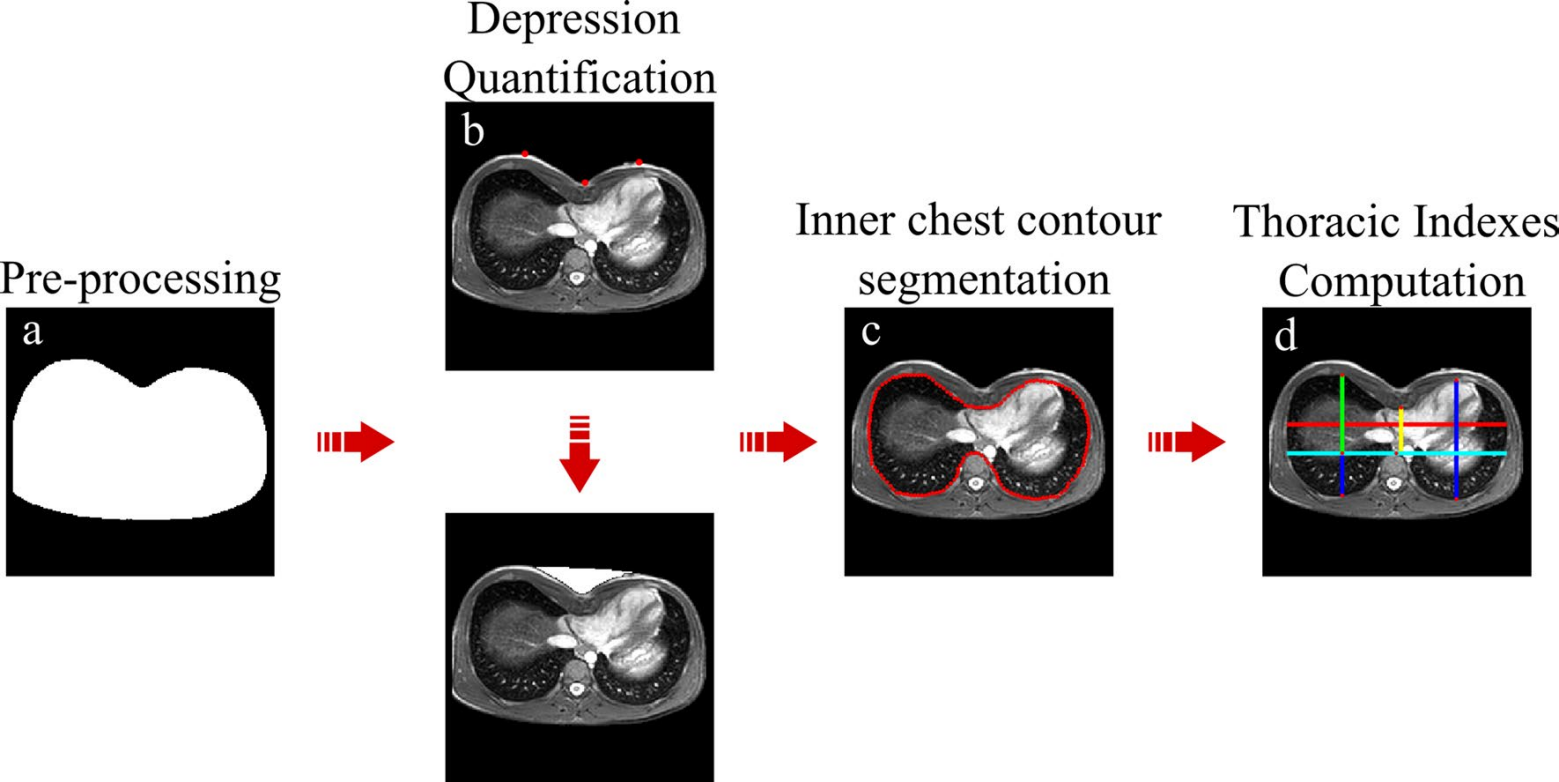
Image analysis framework is composed of four interconnected elements. **a** First module consists in pre-processing of selected slices, that is contrast stretching and cropping on the area of interest. Outcome of this step is a binary mask, used as input for subsequent pipeline. **b** Second module is represented by quantification of the chest depression. Outer chest contour detection serves as a preliminary step for depression computation, quantified as the portion between an elliptic profile and external contour. **c** The latter is exploited for next phase, which is inner chest contour segmentation. This is performed through consecutive sub-steps, which include lung segmentation and similarity between inner and outer wall contour. **d** Final outcome of this pipeline allows to obtain thoracic indexes on the reference slice as well as new volumetric marker. All these measures are saved in a Microsoft Excel® file per each subject

Current framework is organized in four interconnected modules summarized in Fig. 1: Pre-processing, Depression quantification, Inner chest contour segmentation and Thoracic indexes computation. The software code has been developed in MATLAB® 2020a (https://it.mathworks.com/), running in Windows 10.

As a preliminary step for subsequent analyses, a range of slices of interest must be selected, including the slice of maximal sternal depression, on which measurements for PE indices are usually performed in clinic. Indeed, not all axial images acquired are useful for our analysis, but only the ones in which the chest depression and the lungs are clearly visible and thus could be quantified. Of course, this excludes marginal slices at the beginning and at the end of the scan, where the amount of depression is negligible.

This step has been implemented through a Graphical User Interface for selecting range of slices, slice for PE indexes computation, as well as patient’s sex (Additional file 1: Fig. 1).

### Pre-processing

In order to improve low contrast inherent to MR images, firstly we perform a contrast adjustment by remapping the values of the input intensity to fill the entire intensity range. Then, we focus exclusively on chest district by excluding arms placed at the borders of images, due to the small dimension of chest in pediatric patients. This is obtained by defining a proper mask, based on subject’s thorax morphology (Additional file 1: Fig. 2).

**Fig. 2:**
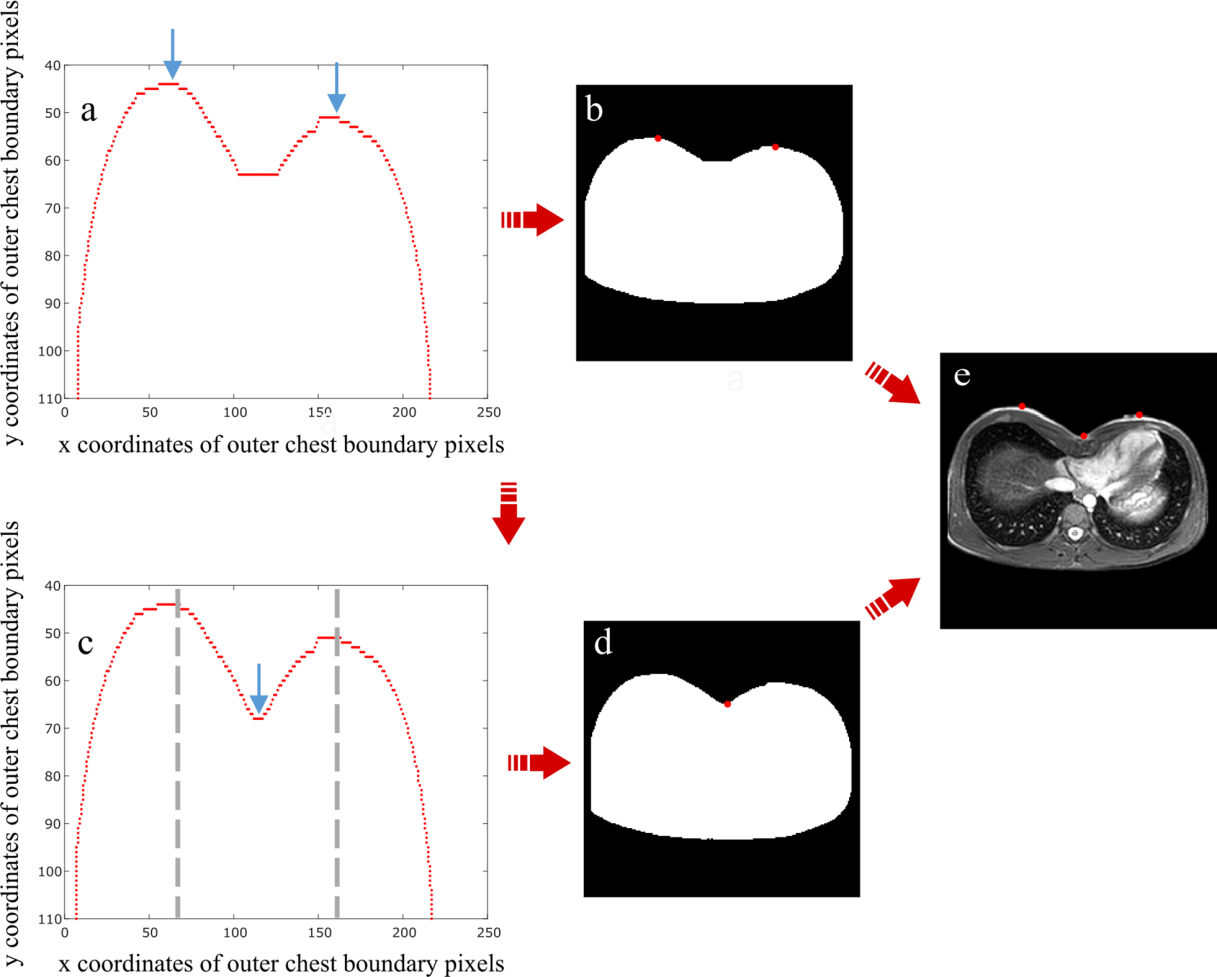
Outer chest contour detection. **a** Plot of upper half of outer chest boundary pixel coordinates after morphological operations, among which research of the two maximum points, shown with light blue arrows, is performed. **b** Binary image with the two maximum points in red, identified after morphological operations. The image shows as the latter modifies the minimum position. **c** Plot of upper half of outer chest boundary pixel coordinate before morphological operations. Between the two already identified maximum points (dashed gray vertical lines), minimum point research is performed. **d** Binary image with the minimum point in red, identified before morphological operations. **e** Grey-scale image with the two maximum and the minimum points in red

### Depression quantification

This module has the goal to quantify the depression, based on a volumetric study. Indeed, rather than evaluating the depression on a single slice, as traditional radiological indices commonly adopted in clinical practice do, we propose to analyze multiple slices in order to measure the depression volume. The idea is to identify the two maximum and the minimum points of the outer chest contour for each slice considered and thus define an elliptic curve between the two maximum points to correct the depression and simulate the normal chest, in absence of PE malformation. The difference between the chest image before and after image correction gives the amount of the depression.

### Analysis of outer chest contour

First of all, the algorithm turns the grey-scale image into a binary image, by applying a manual threshold (T = 0.1) to separate the foreground from the background pixels. Indeed, this value proves to apply for all examined subjects. Then, we get exterior boundaries of chest in terms of Cartesian *x* and *y* coordinates through morphological gradient operators in order to identify the two maximum and the minimum points of outer chest contour. This is just a rough detection of maximum points, since binarization may alter upper profile of images. We thus resort to morphological operators of closing to correct upper image boundaries. By only considering the upper half of outer chest boundary, the algorithm exploits the spatial discontinuities of boundary pixels along *y* direction in order to identify the two maximum points (Fig. 2a,b). As regards the minimum point, we use boundary pixel locations before morphological operations and find it as lowest peak in the range of *y* coordinates between the two already identified maximum points (Fig. 2c–e).

### Depression volume

By analyzing MR images of chest in normal patients, we have noticed that the best curve, representing the chest morphology between the two maximum points of outer chest contour, could be a roto-translated ellipse, whose points are calculated as follow:

$$\begin{aligned} & x = \frac{{x_{1} + x_{2} }}{2} + a*cos\left( t \right)*cos\left( \alpha \right) - b*sin\left( t \right)*sin\left( \alpha \right) \\ & y = \frac{{y_{1} + y_{2} }}{2} + a*cos\left( t \right)*sin\left( \alpha \right) - b*sin\left( t \right)*cos\left( \alpha \right) \\ \end{aligned}$$

where:

- *(x, y)*: coordinates of ellipse points
- *(x*_*1*_*, y*_*1*_*)*: coordinates of right vertex of major axis
- *(x*_*2*_*, y*_*2*_*):* coordinates of left vertex of major axis
- *a*: semi-major axis
- *b*: semi-minor axis, found as $b=a*\sqrt{1-{ e}^{2}}$
- *e*: ellipse eccentricity
- *t*: variation angle of ellipse points, defined between 0 and π (half ellipse)
- *α*: rotation angle, defined as the angle between the horizontal line and major axis

Eccentricity (*e*) and positions of right and left vertices of major axis ((*x*_*1*_, *y*_*1*_) and (*x*_*2*_, *y*_*2*_)) represent the parameters we have modified in order to simulate the profile of outer chest contour in normal patients. After several tests, *e* has been set to 0.99.

**Fig. 3:**
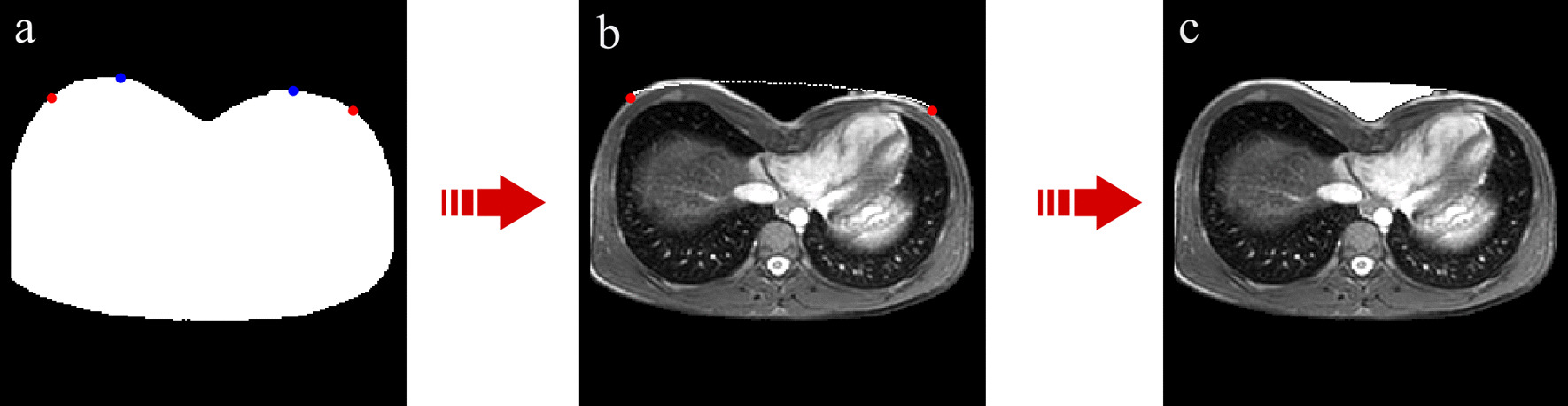
Automatic procedure for depression area filling. **a** Binary image with two maximum points of outer chest contour in blue and two vertices of ellipse major axis in red, after operation of lowering. **b** Grey-scale image with elliptical curve of correction. **c** Grey-scale image with missing chest area, caused by PE malformation, in white

Regarding the position of major axis vertices, we have separated patients based on their sex. Indeed, anatomical differences between male and female forced us to deal with the depression issue in a distinct way. By analyzing normal chest images of male subjects, we were able to find a unique method to define the position of major axis vertices. Specifically, the algorithm identifies *y* coordinates (*y*_*1*_ and *y*_*2*_) by lowering the position of two maximum points of outer chest contour by a constant value, while the x coordinates (× 1 and × 2) are found by searching the most extreme points at the same y coordinates (Fig. 3a).

This correction method does not work in female patients, because the presence of breast rises the position of major axis vertices by causing a wrong depression correction. As among female subjects there is a high variability in chest shape due both to age and differentiated anatomical growth, it is impossible to define a single correction method for the depression. For these reasons, we decided to exclude female patients from depression quantification analysis, while including them in the automatic computation of standard indexes.

After the ellipse has been obtained, the algorithm finds the indices corresponding to *x* and *y* ellipse coordinates in the image matrix and adds pixels to the binary image of chest in these specific locations. Consequently, the depression is filled by applying a morphological operation of closing using a disk as structural element (Fig. 3b).

The depression area is thus calculated as the difference between the image before application of morphological operators and the one after correction with the elliptical curve. Finally, the depression volume is computed by summing the volumes obtained for each slice, resulting from the product of depression areas by ‘slice thickness’ image DICOM attribute. After repeating this operation for each slice, the algorithm is able to represent on grey-scale images how the normal morphology of chest should be (Fig. 3c).

### New pathological marker computation

The absolute value of depression volume cannot be used as a pathological marker since it is strongly dependent on chest dimension and on the number of slices considered for its computation, which is different from patient to patient. Thus, we decided to normalize it on the thorax volume after the correction, as it simulates the ‘normal’ condition of the chest. Specifically, the algorithm quantifies the correct chest volume in the same way as for depression volume by considering the binary image after depression correction. The new pathological marker, that we named Volumetric Correction Index, is defined as follow:

$$Volumetric\,Correction\,Index= \frac{depression\,volume}{correct\,chest\,volume} * 100$$

Therefore, the new index proposed represents the percentage of depression that must be corrected in PE patients.

### Inner chest contour segmentation

This module aims at detecting the inner contour of the chest, fundamental for PE indexes calculation. If this task is difficult in CT images, where the attenuation coefficients of the heart and the chest-wall are quite close, it is even more challenging in MR images, where different chest regions often have a high similarity in terms of grey levels.

Therefore, in order to simplify the segmentation process, we designed this module by subdividing it in consecutive steps, as described in the following sections.

Firstly, the algorithm isolates the inner chest portion by exploiting lung segmentation and similarity between the inner and outer wall contour. Then, it excludes the vertebral body by thresholding method. Finally, it corrects the errors in the detection of inner chest contours through a comparison among consecutive slices. For this analysis step, we opted for working on a limited number of slices, by excluding the ones preceding the slice selected for index computation. Indeed, the remaining range of slices ensures an easily implementable segmentation of inner chest region thanks to an optimal lung-background contrast.

### Lung segmentation

In view of performing segmentation of the lungs, we used histogram analysis for identification of the correct threshold. The grey-level histogram of a MR image is characterized by a high variability, both across subjects and across slices within the same patients, in peaks’ shapes corresponding to the lungs and to cardiac structures and thorax tissue, respectively. For this reason, Otsu thresholding technique [34], the standard approach for histogram partitioning, does not perform well due to its inability to correctly separate bimodal histograms when the two classes are very different in size. Therefore, we developed a method to automatically partition a grey-level histogram, by adapting an algorithm presented by [35]. The idea proposed by this study, that we applied to our problem, is to locate the concavity between the two principal peaks in the curve representing the image histogram by maximizing divergence between the histogram and a Gaussian fit.

After computing the histogram of the grey-scale image in continuous form, the algorithm defines an auxiliary curve *P(x)* on the same grey-level range of the histogram *H(x)*. We assumed *P(x)* as a normal distribution, with mean given by *µ*, the average gray-level of *H(x)*, and the corresponding variance given by *σ*^*2*^. We also considered *P(x)* and *H(x)* to have an identical area *α* under their curves. Given that *x*_*min*_ ≤ *x* ≤ *x*_*max*_, *P(x)* is defined as:$P(x) = \frac{\alpha }{z} G(x)$where:

- $$G(x)= \frac{1}{\sqrt{2\pi {\sigma }^{2}}}exp \left(\frac{{-(x - \mu )}^{2}}{2{\sigma }^{2}}\right)$$
- $$z (x) =\sum_{x={x}_{min}}^{{x}_{max}}G (x)$$
- $$\alpha =\sum_{x={x}_{min}}^{{x}_{max}}H (x)$$
- $$\mu =\frac{1}{\alpha }\sum_{x={x}_{min}}^{x={x}_{max}} x H(x)$$
- $${\sigma }^{2} =\frac{1}{\alpha }\sum_{x={x}_{min}}^{x={x}_{max}} {(x - \mu )}^{2}H(x)$$

As a normal distribution, *P(x)* presents its largest value at *x* = *µ* and has a convex part that goes from *µ − σ* to *µ* + *σ*. The oddity is that the highest peaks of *H(x)* are close to *µ*, the average grey level, such that the concavities surrounded by the highest peaks in *H(x)* are often in contrast with the convex part of *P(x)*. Hence, the line (*l*) that divides the main peaks in *H(x)* can be easily found by maximizing the difference between *P(x)* and *H(x)*, or formally: $l~ = arg\mathop {\max }\limits_{x} ~\left( {{\text{P}}\left( x \right){\text{~}}{-}{\text{~H}}\left( {\text{x}} \right)} \right){\text{~}}$, with $\mu - \sigma \le x\le \mu +\sigma$.

The *l* value corresponds to the threshold separating the two main peaks in image histogram. We implemented this histogram partitioning method in MATLAB and applied it twice in our analysis. First, it is used to separate the chest area from the background. Thus, it analyzes the lower part of histogram, by considering as input the range of *x* values in the low grey-level region. Once found the threshold (*l*_*bg*_) that removes the background from the image, a new grey-level histogram *H’(x)* is generated, considering only the pixels related to the chest. Hence, the method is reapplied to the new data to estimate the correct threshold for the lung segmentation (*l*_*lung*_). Specifically, in order to enhance the threshold search, the algorithm focuses the analysis on the lower part of *H’(x)*, since it corresponds to grey values belonging to lungs (Additional file 1: Fig. 3).

After finding appropriate thresholds for each slice with this strategy, these are used to segment lungs from chest region. Specifically, a mask is created where pixels with intensity above the *l*_*lung*_ are set as white and the remaining ones are set as black. Other segmented elements besides the lungs are removed and morphological operation of closing are applied to smooth edges and fill holes inside the lungs.

### Selection of appropriate slice for indices computation

Finally, before proceeding to inner contour detection, we created an automatic technique to exclusively select the slices where lungs are clearly visible. Indeed, we wanted to exclude from further analysis those slices where inner chest region segmentation could be complex, due to the absence of lungs. Specifically, the algorithm computes the lung area and relates it to the entire chest area. Then, it selects only the slices in which the ratio is greater than 20%. Therefore, if the slice selected for indices calculation shows high similarity in grey values between different chest areas, the algorithm automatically picks the first following slice, where inner chest contour detection can be performed properly.

### Inner chest contour detection

In order to isolate the inner thoracic region, we adapted an algorithm proposed by [36], for the inner curvature detection of CT images. Specifically, they proposed a recursive algorithm that exploits outer wall contour as a starter point for inner contour segmentation, due to similarity in morphology between them.

We identified as algorithm inputs, obtained from previous module, the matrices composed of pixel locations of each lung and the matrix containing pixel locations of outer curvature.

**Fig. 4:**
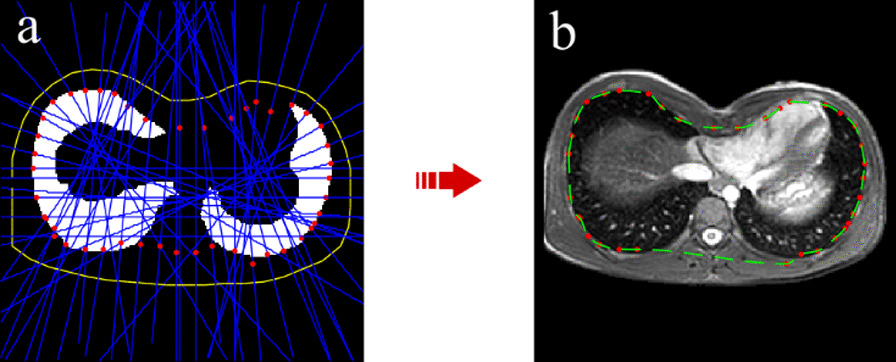
Algorithm for preliminary inner wall contour **a** Binary image representing lung region. Yellow line represents outer chest curvature. In blue there are the perpendicular lines, generated every 12th step. In red there are the intersection points resulting from recursive algorithm. **b** Gray-scale image on which inner mask boundary points are indicated in green, while intersection points found by recursive algorithm in red. This is clearly a rough contour of inner chest, including vertebral body, and thus further corrections are required

The algorithm goes through steps along the outer curvature in clockwise direction until the start point is found again. Every 12 steps the actual point and the point 12 steps before are connected and a perpendicular line in the mid-point of their connection is generated. Then the algorithm finds the intersection point between the perpendicular line and the first point crossed by it on the two lungs. In the area of the binary image where the perpendicular lines do not cross any lungs, a correction of the invalid points generated is necessary. Thus, it calculates for each line the distances between the midpoint and the intersection point and computes their mean value (*µ*_*d*_) and the standard deviation (*σ*_*d*_). We have designed a length filter by defining as invalid the intersection points whose distances from the mid-points are longer than a specific threshold that we identified as 2* µ_*d*_* − σ*_*d*_. All points, corresponding to longer distances than this value, are deleted and replaced by new ones located at the same distances as the previous valid point (Fig. 4a). Once all intersection points have been found, the inner curvature is calculated by an interpolating process. Initially, the algorithm prepares intersection points by separating them in two subsets: the ones related to the upper half of the inner contour and those belonging to the lower part. Additionally, it performs an initial correction, by deleting points whose *y* locations are in discontinuity with *y* positions of neighboring points, in order to avoid the eventual errors made by previous operations. Then it applies a shape-preserving piecewise cubic interpolation method (‘*pchip*’) with a high sampling rate. Finally, we obtain a group of closely spaced points both for superior and inferior half of inner contour. After re-combining them in a unique set of points, we can define the boundary of a mask that isolates the inner thoracic region (Fig. 4b). However, this mask also includes the vertebral body and is not accurate in all the slices, mostly due to bad lung segmentation. For these reasons, it is necessary to improve the inner chest segmentation with further processing.

### Inner chest contour correction

**Fig. 5:**
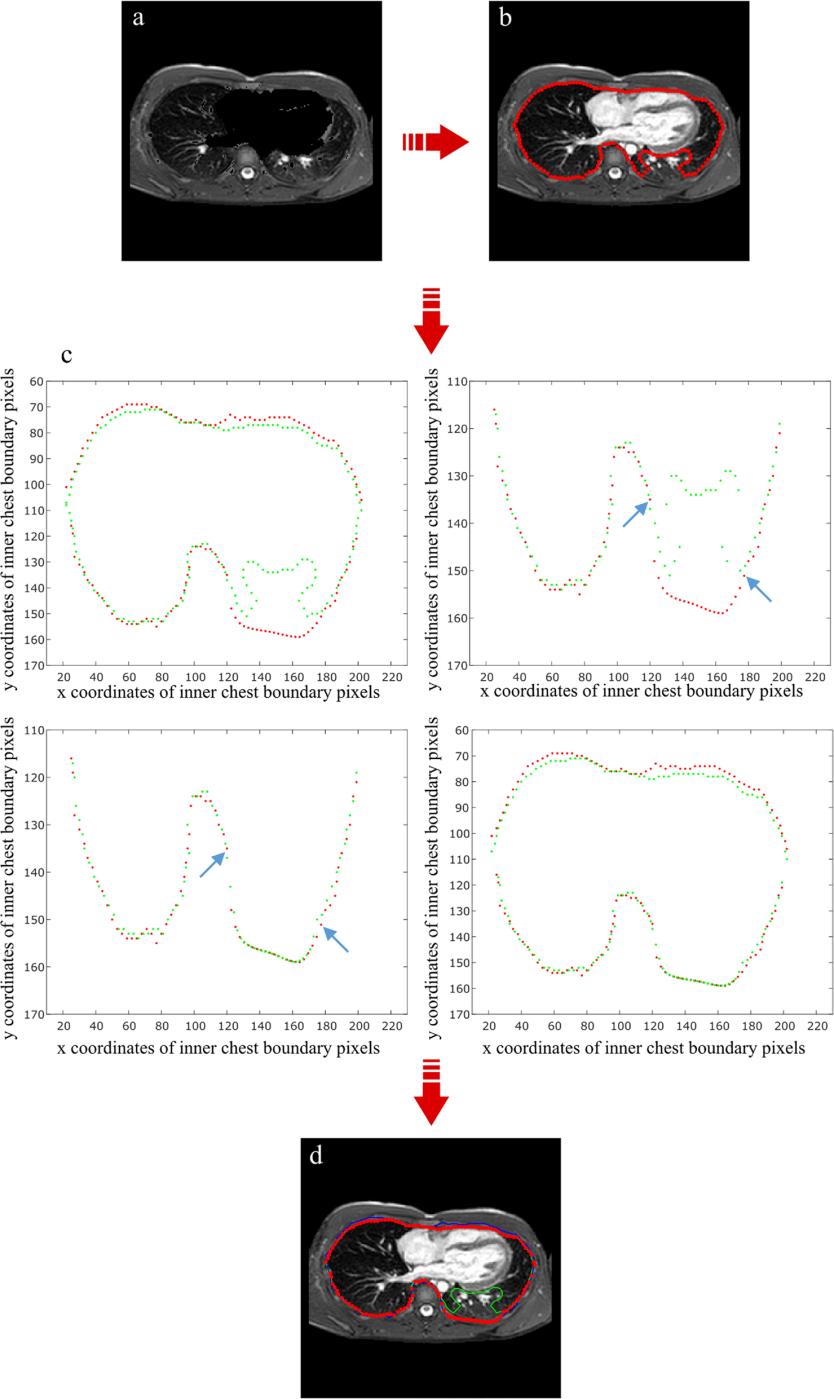
Algorithm for inner wall contour correction. **a** Grey-scale image after masking out cardiac structures in order to create a mask of inner thoracic area. **b** Grey-scale image with inner chest boundary pixel locations in red. The error appears around inferior lung area, that has grey values close to thoracic tissue ones. **c** Plot of *x* and *y* coordinates corresponding to inner chest boundary pixels. In red there is the reference curve used for correction, while in green the curve that need to be corrected by algorithm. Between two blue arrows there are the points resulting from interpolation process that substitute incorrect ones. **d** Inner chest boundary pixel locations after correction are represented in red while those before correction in green; in blue there is inner contour of the reference curve, belonging to previous slice

The first step of inner chest contour correction consists in excluding the vertebral body. For doing so, the algorithm applies a thresholding method by using the inner mask found in previous section as a tool to improve segmentation. After masking out the external chest area, a slice-wise threshold is defined to exclude out heart and other cardiac elements through histogram partitioning method presented in previous section. Indeed, correction is not possible without masking out cardiac district, due to high similarity in grey values between heart and thoracic tissue. Thus, the algorithm returns to original chest image, before the application of inner mask, and assigns to background the just segmented pixels belonging to cardiac structures (Fig. 5a). Then, it is able to separate the inner chest region from the outer chest one, by applying as threshold the same value found for lung segmentation (*l*_*lung*_). To have the inner region as foreground, the complementary image is computed, and some morphological operations are applied to smooth the edges and fill the holes. Finally, we obtain a binary image representing the inner chest region from which to extract boundary pixel locations for each slice.

However, a further correction may be necessary both around the vertebral body, having grey values close both to lungs and to cardiac structure intensities, and around inferior lung area, being often difficult separating pixels belonging to lungs and to thoracic tissue ones (Fig. 5b).

We thus designed a method to correct the inner chest contour by comparing consecutive slices thanks to high similarity in boundary pixel positions belonging to the lower half of inner contour of our interest. At this step, user intervention is required such that correction process starts from a slice where inner contour detection does not present errors. Once first slice is selected, the algorithm starts the pair-wise comparison among adjacent slices in both directions, by taking as reference the points belonging to the contour of slice selected. Thus, the algorithm computes the distance among them and all the points belonging to the contour of the adjacent slice, which is assumed as incorrect. Then it creates a vector that, for each point belonging to the contour to correct, only keeps the minimum distance among all those just computed. It also calculates the maximum value (*d*_*max*_) and the standard deviation (*σ*_*d*_) of all minimum distances. After several tests, we established that the algorithm must continue only if *σ*_*d*_ is greater than 1.8. Additionally, we identified *d*_*max*_ -2* σ_*d*_ as threshold value that separates correct points from incorrect ones. Thus, for each incorrect point, the algorithm finds the range that must be deleted, by identifying its extremities in the nearest points to the correct curve. Then, it replaces them with the points belonging to the correct curve by using a shape-preserving piecewise cubic interpolation method (‘*pchip*’) (Fig. 5c). Once a new curve is obtained, the algorithm proceeds to the next slice, by taking as reference the just corrected contour. Such an algorithm allows to satisfactorily correct errors in segmentations (Fig. 5d).

### Thoracic indexes computation

**Fig. 6:**
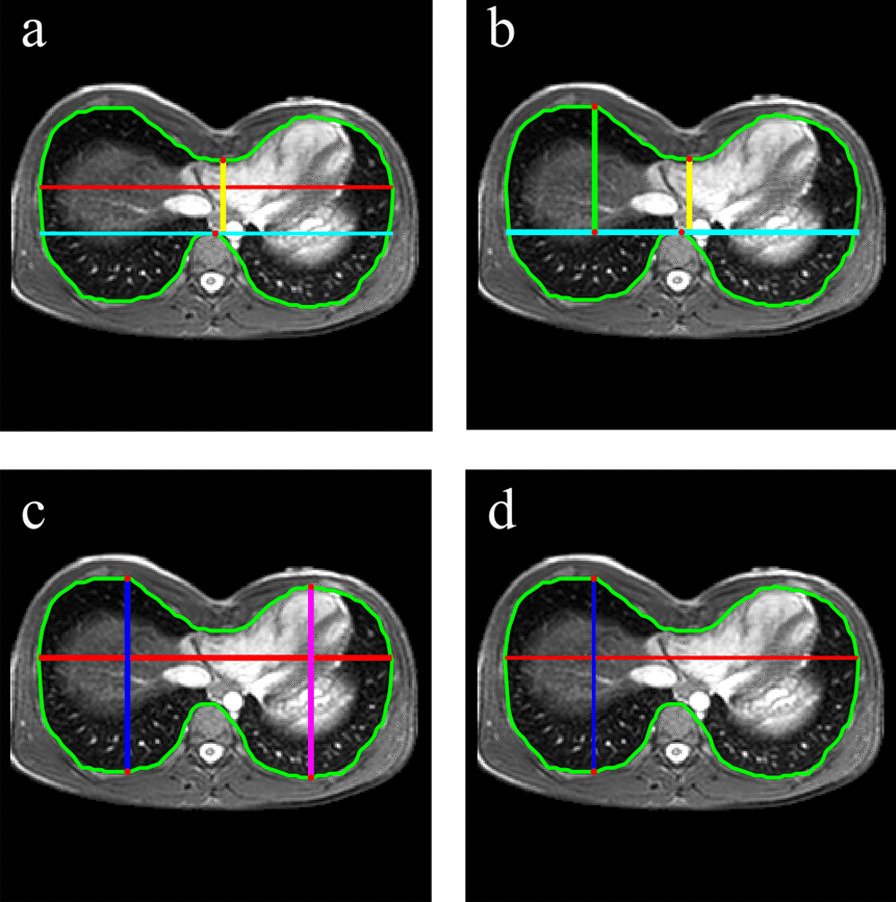
Inner thoracic distances overlaid on slice of maximal sternal depression. **a** Distances useful for iHaller computation: transverse diameter in red, min APd in yellow. In cyan there is the horizontal line at same y position of vertebral body. **b** Distances useful for iCorrection computation: min APd in yellow, max APd in green. In cyan there is the horizontal line at same y position of vertebral body. **c** Distances useful for iAsymmetry computation: right hemithorax APd in blue and left hemithorax APd in magenta. **d** Distances useful for iFlatness computation: right hemithorax APd in blue and transverse diameter in red

This module aims at computing PE indices used by physicians to classify the severity of patients’ malformation. As mentioned above, among multiple thoracic markers, we focused on the severity (Haller index and Correction index) (Fig. 6a, b) and deformity (Asymmetry index and Flatness index) ones (Fig. 6c, d). The algorithm only works on the first slice of images processed in the previous module. Indeed, it corresponds to the slice selected by the user or to the first following one where inner chest contour can be detected.

Once inner distances and thoracic indices are computed, the framework saves their results along with the new pathological marker obtained in Depression quantification module in an Excel® file, located in the same folder as patient’s images. Each quantified distance in following computations has been multiplied by ‘pixel spacing’ attribute to have measures in mm.

### Haller index

Haller index (iHaller) is calculated by dividing the transverse diameter, i.e., the widest horizontal distance of the inside of the ribcage, to the minimum anteroposterior diameter (min APd), i.e., the shorter distance between the vertebral body and the sternum [12].

$$iHaller=\frac{transverse\,diameter}{min\,APd}$$

As regards transverse diameter, the algorithm identifies its first extremity as the point on inner chest contour with minimum *x* coordinate and the second one as the point at its same *y* coordinate. Conversely, the first extremity for measuring min APd corresponds to sternum position. We approximate it as the point with maximum *y* coordinate (*y* values decrease toward the bottom of image) by only considering the range of inner chest contour points between *x* coordinate positions of two maximum points of outer chest contour upper half. Second extremity corresponds to the vertebral body position. It is taken as the point with minimum *y* coordinate by only considering the range of inner chest contour points between position of *x* coordinates of two maximum points of outer chest contour lower half.

### Correction index

Correction index (iCorrection) is calculated by dividing the amount of defect, measured as the difference between the maximum anteroposterior distance, i.e. the maximum distance between the anterior spine and the anterior portion of the chest (max APd) and the minimum anteroposterior diameter (min APd), to the maximum anteroposterior distance (max APd), multiplied by 100 [37].

$$iCorrection=\frac{(max\,APd - min\,APd)}{min\,APd} * 100$$

For max APd computation, firstly, the algorithm draws a horizontal line at the same *y* coordinate of vertebral body position, that is assumed as the anterior spine position. Then, it identifies two points on inner chest contour at the same *x* coordinate of two maximum points of outer chest contour. The latter are assumed as the positions of right and left anterior portion of the chest. Thus, for each point it computes the distances between them and the horizontal line and gets the maximum diameter between the two distances.

### Asymmetry index

Asymmetry index (iAsymmetry) is calculated by dividing the longest anteroposterior distance of the right chest wall (right hemithorax APd) to the longest anteroposterior distance of the left chest wall (left hemithorax APd), multiplied by 100 [38].

$$iAsymmetry=\frac{right\,hemithorax\,APd}{left\,hemithorax\,APd} * 100$$

Right hemithorax APd’s extremities are identified as the points on inner chest contour at the same *x* coordinate of first maximum point, as it is located in right hemithorax. Right hemithorax APd’s bounds are identified as the points on inner chest contour at the same *x* coordinate of second maximum point, as it is situated in left hemithorax.

### Flatness index

Flatness index (iFlatness) is computed by dividing the transverse diameter of the thorax to the longer of the two maximum anteroposterior diameters of the right (right hemithorax APd) and left hemithorax (left hemithorax APd) [13]. As all the distances have been already found, the algorithm can proceed with Flatness index computation, as follow:

$$iFlatness=\frac{transverse\,diameter}{max\left(righthemithoraxAPd;\,lefthemithoraxAPd\right)}$$

### User’s correction

As mentioned above, the algorithm does not always perform indices computation on the same slice selected by user, due to its inability to segment images where different chest areas have similar grey values. In these cases, it selects the first following slice, where inner chest contour detection can be performed. We noticed that by going through consecutive slices some inner distances maintain their value constant, while others, specifically min APd and max APd are more likely to vary. For this reason, in the algorithm we add the possibility of user’s intervention when the slice is different from the one selected. Specifically, the user is asked to insert two points on the image, useful for min APd and max APd calculation: sternum position and vertebral body position. Thus, the algorithm recomputes the indices by considering the modifications on these two inner distances. Finally, two sets of results are obtained: the ones calculated on the slice picked by the algorithm and those obtained on the same slice selected after correction of sternum and vertebral body points.

## Modules validation

Current methodological framework has been developed from a small subset counting 5 subjects. In order to test the overall quality of our algorithm, we extended its application to other 45 pediatric patients affected by Pectus Excavatum from Gaslini Children’s Hospital, in Genoa, for a total of 50-subjects dataset.

Additionally, two expert radiologists manually performed double-blind thoracic indices computation, as they routinely do in the clinical setting. The group of patients consisted in 41 males and 9 females aged 13.5 ± 2.78 (mean ± SD), age range 5–18 years. Each of them underwent MRI examination, in order to establish the severity of malformation and thus the best treatment strategies. MRI examinations were performed on a 1.5 Tesla MR scanner (Achieva, Philips Healthcare, Cleveland, OH, USA), equipped with 66 mT/m gradients (maximum), a slew rate of 180 mT/m/msec (maximum) and a 32-element cardiac phased-array coil for signal reception and cardiac synchronization (with “retrospective gating” technique). Our MRI protocol borrowed cardiac gating and breath-holding techniques as well as specific sequences from CMRI, in order to overcome motion-related artifacts and to inspect with further detail cardiovascular morphology. The MR acquisition setting thus included scout images and Steady State Free Precession (SSFP) images in axial, coronal and sagittal planes, acquired at the end of expiratory phases. Specifically, the SSFP sequence was a Gradient-Echo sequence, named Balanced Turbo Field Echo-Breath Hold (BTFE-BH). Total scanning time was 5–8 min approximately.

## Results

Out of 50, just three subjects (2 male and 1 female) have been excluded from our analysis since characterized by extremely low contrast images that the algorithm could not process. We can thus conclude that, provided sufficient contrast in the input raw image, proposed method mantains its reliability and accuracy for the whole cohort ander analysis.

Our image processing pipeline has then been quantitatively evaluated through comparison with manual procedure. As mentioned in previous section, the slice selected by user for indices computation is often difficult to segment due to similar grey values of different thoracic regions, so that algorithm automatically picks the first following slice, where inner chest contour detection can be performed. Specifically, out of the 47 patients processed by algorithm, the latter was able to use the same slice as the one selected in 24 patients, while in the remaining it selected another scan. We thus separated the patients in two groups, depending on whether the indices computation was performed on the same slice selected by user (group 1) or it was executed on a different slice picked by the algorithm (group 2).

### Automatic framework agrees with manual procedure for indexes computation

In the absence of a ground truth to test performance against, the accuracy of the thoracic indices resulting from algorithm was evaluated by comparing them to results obtained by manual measures performed by two expert radiologists (through a double-blind analysis).

Table 1 shows the results of patients belonging to group 1. Results of inner thoracic distances show a good agreement between measures obtained by the 2 readers and the algorithm. Naturally, the difference is higher by comparing manual results to automatic ones, as it is shown by a greater mean standard deviation. We can notice that transverse diameter, min APd and max APd are computed by the algorithm in a comparable way as results obtained manually. Contrariwise, right hemithorax APd and left hemithorax APd are characterized by a higher variability that, however, is stronger also between the 2 readers. Obviously, inner distances affect the results of thoracic indices. Specifically, we notice that Haller index and Flatness index results are comparable, whereas the differences between manual and automatic computation increase by considering Correction index and Asymmetry index.

**Table 1:** Average inner thoracic distances and thoracic indices along with relative mean standard deviation between 2 readers and mean standard deviation (std) among readers and algorithm in case of appropriate user selection of main slice for indices computation

	Reader 1	Reader 2	Algorithm	Std between 2 readers	Std among readers and algorithm
Thoracic distances (cm)					
Transverse diameter	24.1 ± 2.6	24.2 ± 2.6	24.4 ± 2.9	0.17	0.30
Min APd	4.9 ± 1.3	5.0 ± 1.4	5.1 ± 1.4	0.18	0.30
Max APd	7.2 ± 0.94	7.6 ± 0.93	7.5 ± 0.95	0.31	0.34
Right hemithorax APd	12.4 ± 1.2	12.6 ± 1.3	12.2 ± 1.2	0.24	0.37
Left hemithorax APd	12.3 ± 1.2	12.6 ± 1.1	11.9 ± 1.2	0.30	0.47
Thoracic indices					
Haller index	5.3 ± 1.9	5.3 ± 1.9	5.1 ± 1.6	0.31	0.34
Correction index (%)	32.5 ± 13.9	35.2 ± 14.1	32.6 ± 13.6	2.7	3.6
Asymmetry index (%)	101.7 ± 6.7	99.8 ± 5.2	102.8 ± 8.2	2.2	3.2
Flatness index	1.9 ± 0.19	1.9 ± 0.17	2.0 ± 0.20	0.038	0.057

### Inner thoracic distances and thoracic indices in case of matching slice selection

Regarding the results belonging to group 2, the measurements obtained from the algorithm were performed on a different slice compared to the one analyzed by the 2 readers. As we mentioned above, we noticed that some distances remained almost constant by measuring them on consecutive slices. Contrariwise, two distances, specifically min APd and max APd, showed more variability among consecutive slices. Consequently, we decided to apply a correction factor to these measurements, to be able to compare the algorithm results to the ones computed by the readers. Specifically, as we observed that algorithm tends to overestimate both min APd and max APd, we subtracted to them a corrective factor that we identified as the mean standard deviation between readers and algorithm (0.50 in both cases). Table 2 shows the results of patients belonging to group 2, after the just mentioned correction of the two inner distances. The same considerations made for inner thoracic distances results belonging to group 1 apply also in this case. However, by observing thoracic indices results, we notice a higher variability among readers and algorithm than the one found in Table 1. The reason is mainly due to the use of a different slice for indices computation. Furthermore, there are more severe cases of PE among patients belonging to this group. This aspect could be another cause for the higher variability in indices, specifically Haller index. Indeed, we noticed that differences among reader and algorithm results increase when the min APd assumes low values, as it is placed at the denominator in the index calculation formula. Thus, variability is higher for high Haller indices rather than lower ones.

**Table 2:** Average inner thoracic distances and thoracic indices along with relative mean standard deviation (std) between 2 readers and mean standard deviation among readers and algorithm in case of failed user selection of main slice for indices computation

	Reader 1	Reader 2	Algorithm	Std between 2 readers	Std among readers and algorithm
Thoracic distances (cm)					
Transverse diameter	23.2 ± 1.6	23.3 ± 1.6	23.5 ± 1.7	0.06	0.26
Min APd	3.9 ± 1.4	4.0 ± 1.6	4.2 ± 1.6	0.16	0.36
Max APd	6.5 ± 0.85	6.9 ± 0.83	6.8 ± 0.94	0.25	0.35
Right hemithorax APd	11.3 ± 1.1	11.5 ± 1.2	11.2 ± 1.4	0.17	0.39
Left hemithorax APd	11.9 ± 0.96	12.0 ± 1.0	11.7 ± 1.1	0.14	0.33
Thoracic indices					
Haller index	7.2 ± 4.0	7.2 ± 4.3	7.0 ± 4.6	0.23	0.63
Correction index (%)	42.1 ± 17.9	42.5 ± 19.9	39.1 ± 18.8	2.5	4.7
Asymmetry index (%)	95.1 ± 5.5	96.0 ± 6.8	96.5 ± 8.1	1.4	4.0
Flatness index	2.0 ± 0.16	1.9 ± 0.17	2.0 ± 0.16	0.023	0.050

### Inner thoracic distances and thoracic indices in case of not matching slice selection

Additional file 1: Fig. 4 displays scatter charts representing comparison among results obtained by readers and algorithm for each thoracic index, belonging to patients of group 1.

Finally, the average time necessary to perform traditional indices computation on a single patient was 50 s and 3 min 45 s for automatic (on a standard Windows workstation with i7-core and 8 GB RAM) and manual processing, respectively. However, if radiologists are beginners, the time could significatively increase, even rising to twice the value indicated for manual computation.

### New volumetric index as a promising marker of PE severity

**Fig. 7:**
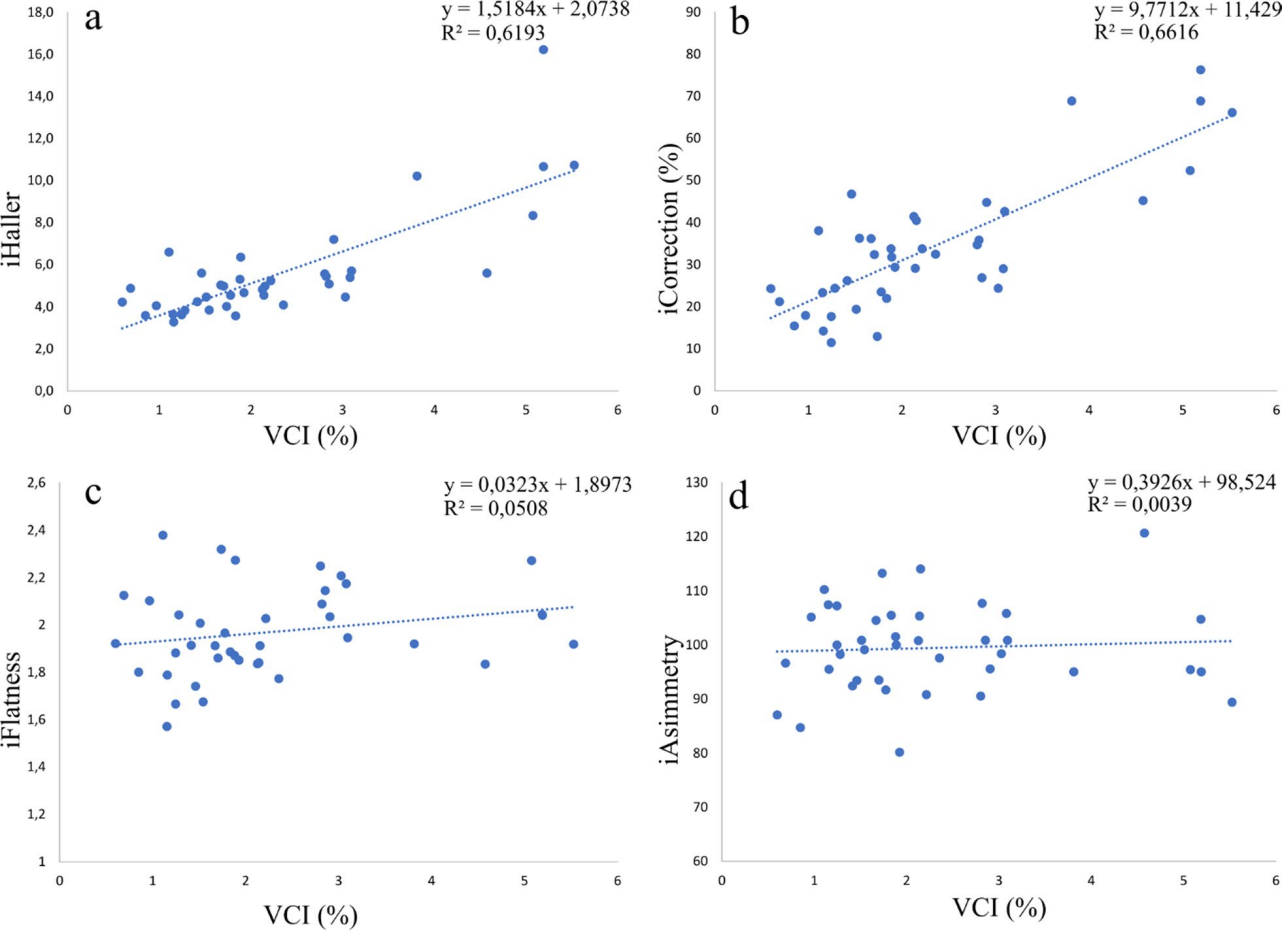
Linear relationship of new volumetric index with existing clinical markers **a** Linear Regression between VCI and iHaller. **b** VCI and iCorrection. **c** VCI and iFlatness. **d** VCI and iAsymmetry. Estimates for the slope and intercept of the linear equation as well as R^2^ are reported for each measure

Finally, the new index calculated by our algorithm exclusively on male subjects (n = 39), named VCI, was compared to all the thoracic indices, in order to evaluate its feasibility for quantitative evaluation of PE. Specifically, we calculated statistical Pearson correlation between VCI and other traditional indices, as shown in Table 3. Furthermore, scatter plot analysis between new pathological marker and other indices are shown in Fig. 7.

**Table 3:** Result of Pearson correlation between traditional indices and new pathological marker computed by algorithm

Indices comparison	Pearson correlation coefficient
iHaller – VCI (%)	0.79
iCorrection (%) – VCI (%)	0.81
iAsymmetry (%) – VCI (%)	0.062
iFlatness – VCI (%)	0.22

The results of correlation show a very low correlation between VCI marker and Asymmetry index and Flatness index (Fig. 7a, b). We expected this behavior since both indices do not quantify the severity of depression but the degree of chest asymmetry and flatness. Contrariwise, the correlation increases with severity indices, i.e. Haller index, and slightly more with Correction index, that are the most used by physicians to assess the PE malformation in a quantitative way. As it is shown in Fig. 7c, small depressions (low VCI) correspond to low Haller indices. However, if Haller index begins to increase the linear relation tends to disappear, as the same iHaller corresponds to different degrees of depression. Same behavior is visible in Fig. 7d that shows the correlation between VCI and iCorrection. However, it should be noted that severe cases of PE with high iHaller and iCorrection are few among all the patients analyzed. Thus, the lack of linear tendency could be caused by a limited number of cases with high degree of PE severity.

### Person correlation between Volumetric Correction Index and other traditional indices

See Table 3.

## Discussion

We introduce a set of tools to aid the pre- and post-surgery assessment of PE patients. We opted for developing this algorithm within an existing software rather than a new stand-alone tool in order to ensure later extensibility across different centers.

The set of algorithms have been tested both qualitatively and quantitatively through a cohort of 50 pediatric patients with varying age, sex and disease severity. In our study, we were able to show that automatic results obtained by our algorithm are comparable with the ones manually computed by expert radiologist. The proposed algorithm offers different advantages First of all, it gives physicians an accurate tool not subjected to individual interpretation or errors and represents a useful support in establishing proper treatment decision, including the need for surgical correction of malformation. Moreover, it ensures a faster processing time compared to manual measurements, which gets relevant in case of large datasets and radiologists with limited experience. Furthermore, for now limited to the male subset of our cohort, we suggested a new pathological marker to better quantify the depression caused by PE: the VCI. Indeed, indexes used so far are based on linear measurements of chest diameters, but they do not evaluate the chest in the tridimensional aspect of the deformity, which has clinical implications. A patient with a deep but very localized PE could have a worse Haller index or Correction index than another patient with a less severe but more extended PE, even if the real impact of the deformity and the compression on lung and heart could be globally worse in the second patient, due to the diffuse PE. Therefore, an index which considers all the missing volume of the thorax and not only measures the severity of PE at a single level could overcome the limits of traditional thoracic indices, such as the dependence on the slice selected for measurements or chest shape. Theoretically, it could also have a better clinical correlation than the current indexes. Nevertheless, further investigations are required to prove the clinical relevance of VCI and incorporate it among the clinical and radiological parameters considered in the decision regarding surgical indication.

Our work has made a relevant contribution to the literature. Indeed, the other studies focused on automatic or semi-automatic quantification of the markers of chest-wall deformity are exclusively limited to CT scans [14, 15, 36, 39, 40].

The peculiarity of our algorithm is that it works on MR images. The adoption of MRI in the evaluation of this condition is relatively recent since, despite their non-invasiveness, MRI scans are more complex to process with automatic segmentation methods than standard CT ones [21, 23, 29, 30].

By analyzing patient-wise results, we could notice that accuracy of algorithm outcomes is strongly dependent on quality of MR images acquired. Thus, optimization of acquisition setting would lead to higher-quality images and thus improve pipeline’s performance without the need of further corrections beside main modules. This improvement may also allow to properly handle with the quantification of depression volume for female patients.

Indeed, one current limitation of this study is exclusion of female patients from computation of newly proposed VCI marker, given the higher variability in chest shape caused by differential breast growth. A similar situation may apply for overweight patients with relevant gynecomastia. However, both target patients substantially represent outliers for this kind of condition, mainly affecting under- or normal-weight male adolescents [41–43]. As a result, current algorithm already performs successfully for most PE candidates, being other cathegories statistically negligible in their amount.

Another possible limitation to the accuracy in calculation of VCI is the thickness of subcutaneous fat tissue in pectoral region. However, as anticipated, the vast majority of PE patients are very slim so the influence of the subcutaneous tissue is in our opinion neglectable in most patients. Calculating VCI at the level of cartilage and bones instead of the inner skin level could potentally overcome this limitation.

One last potential future development of present algorithm may include the computation of cardiac indices. Specifically, it may be useful to develop a new method for quantification of cardiac compression caused by PE malformation, in order to compare it with the new pathological marker proposed.

## Conclusions

In this work we present a piece of software specifically designed to support radiologists in diagnosis and best personalized treatment choice for patients affected by PE condition. Indeed, our study proved its reliability and robustness in processing a discrete number of MR images ranging across different degrees of PE severity. Our tool significantly eases assessment of pathology by improving accuracy of thoracic distances and subsequent clinical indexes beyond subjectivity inherent to manual intervention, and by reducing the time required for computation of these markers as well.

Given the relatively high incidence of this disease (1:400 live births), disposing of a novel semi-automatic supportive tool enriched with an easily extensible, user-friendly interface may have a substantial clinical impact. Finally, formulation of a new relevant marker for PE scoring paves the way for exploring new strategies for PE assessment.

### Availability and requirements

- Project name: e.g. PE_pipeline
- Project home page: https://github.com/rosella1234/PE_pipeline/
- Operating system(s): Platform independent
- Programming language: MATLAB®
- Other requirements: none
- License: e.g. MIT
- Any restrictions to use by non-academics: e.g. licence needed for MATLAB®

## Supplementary Information

**Additional file 1: Figure 1.** Graphical User Interface for slices selection. **Figure 2.** Image border correction. **Figure 3.** Histogram partitioning for lung segmentation. **Figure 4.** Comparison between double-blind manual measurements and automatic algorithm for computation of thoracic indexes for patients of group 1 a.

## Data Availability

The dataset supporting the conclusions of this article is not publicly available due to privacy restrictions of clinical data imposed by the Gaslini Hospital’s administration but is available from the corresponding author on reasonable request. The Software has been released under MIT License. Code for our pipeline is available at https://github.com/rosella1234/PE_pipeline. Current software has been designed and tested under MATLAB® 2020a. A proprietary license is required for using MATLAB®.

## Abbreviations

CT: Computed tomography
CMR: Cardiac magnetic resonance
DICOM: Digital imaging and communications in medicine
GUI: Graphical user interface
iAsymmetry: Asymmetry index
iCorrection: Correction index
iFlatness: Flatness index
iHaller: Haller index
max APd: Maximum anterior–posterior diameter
min APd: Minimum anterior–posterior diameter
MRI: Magnetic resonance imaging
PE: Pectus excavatum
VCI: Volume Correction Index
