# Supplementary material for: A new tool for assessing Pectus Excavatum by a semi-automatic image processing pipeline calculating the classical severity indexes and a new marker: the Volumetric Correction Index
Source: BMC Med Imaging. 2022 Feb 20;22:30. doi: 10.1186/s12880-022-00754-0 (PMC8859892; doi:10.1186/s12880-022-00754-0)
Supplement: Supplementary file 1 — Additional file 1: Figure 1. Graphical User Interface for slices selection. Figure 2. Image border correction. Figure 3. Histogram partitioning for lung segmentation. Figure 4. Comparison between double-blind manual measurements and automatic algorithm for computation of thoracic indexes for patients of group 1 a. [file 12880_2022_754_MOESM1_ESM.docx]

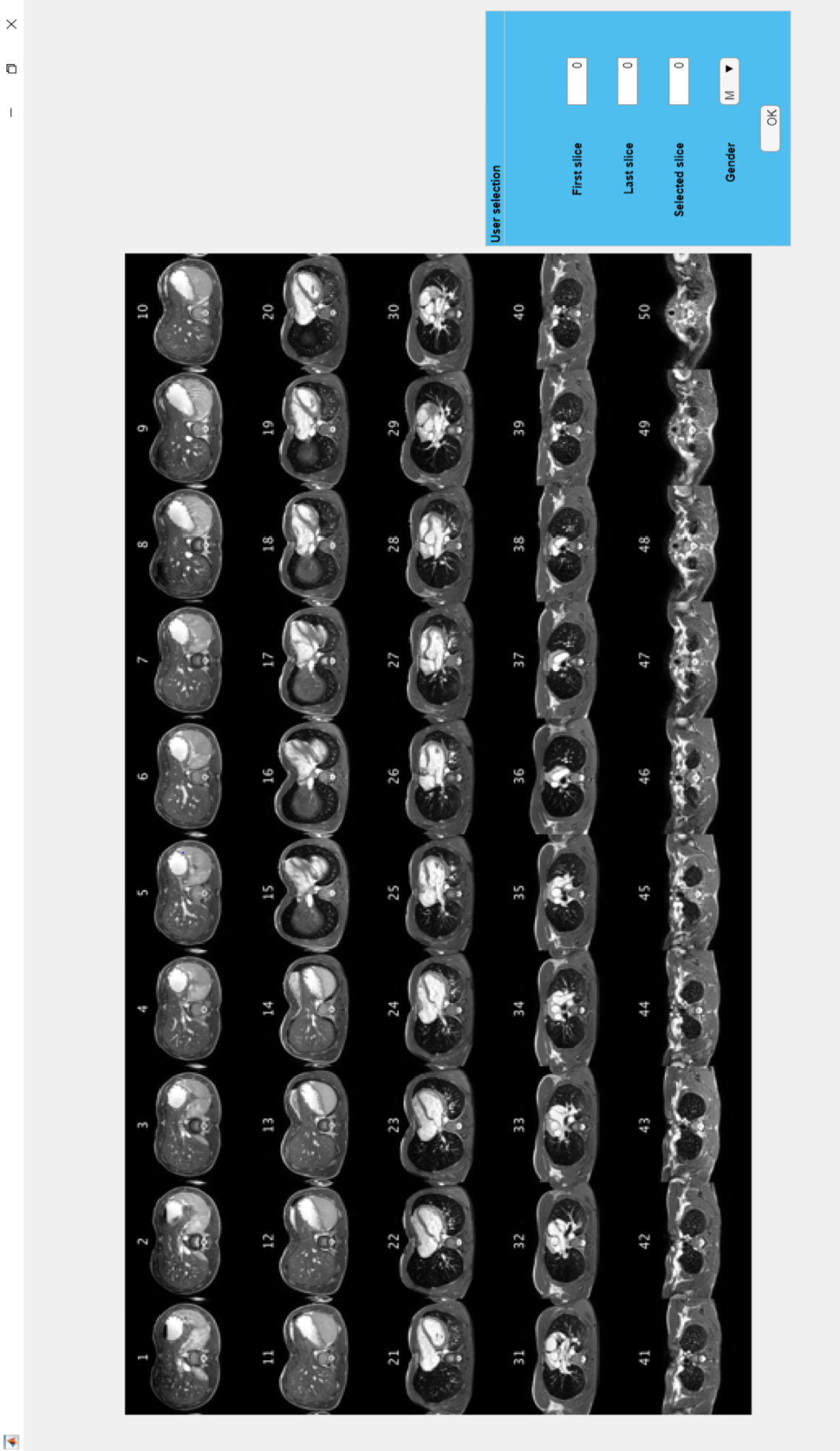


**Additional Figure 1. Graphical User Interface for slices selection.** It allows the user to choose MR slices among the axial images acquired. Slices of interest are required to have a sufficient contrast between different anatomical structures and are chosen across the slice of maximal sternal depression, on which mostly of measurements for PE indices calculation are performed. The MR images, which are in DICOM format, are loaded by specifying the folder path. Then, all axial images belonging to the patient analyzed are visualized simultaneously to ease selection. Additionally, DICOM information are stored in order to obtain data related to image acquisition process. Finally, the algorithm requires the radiologist’s intervention since user is asked to select: the range of consecutive slices where the depression needs to be quantified, the slice of maximal sternal depression, where indices calculation has to be performed, patient’s sex.


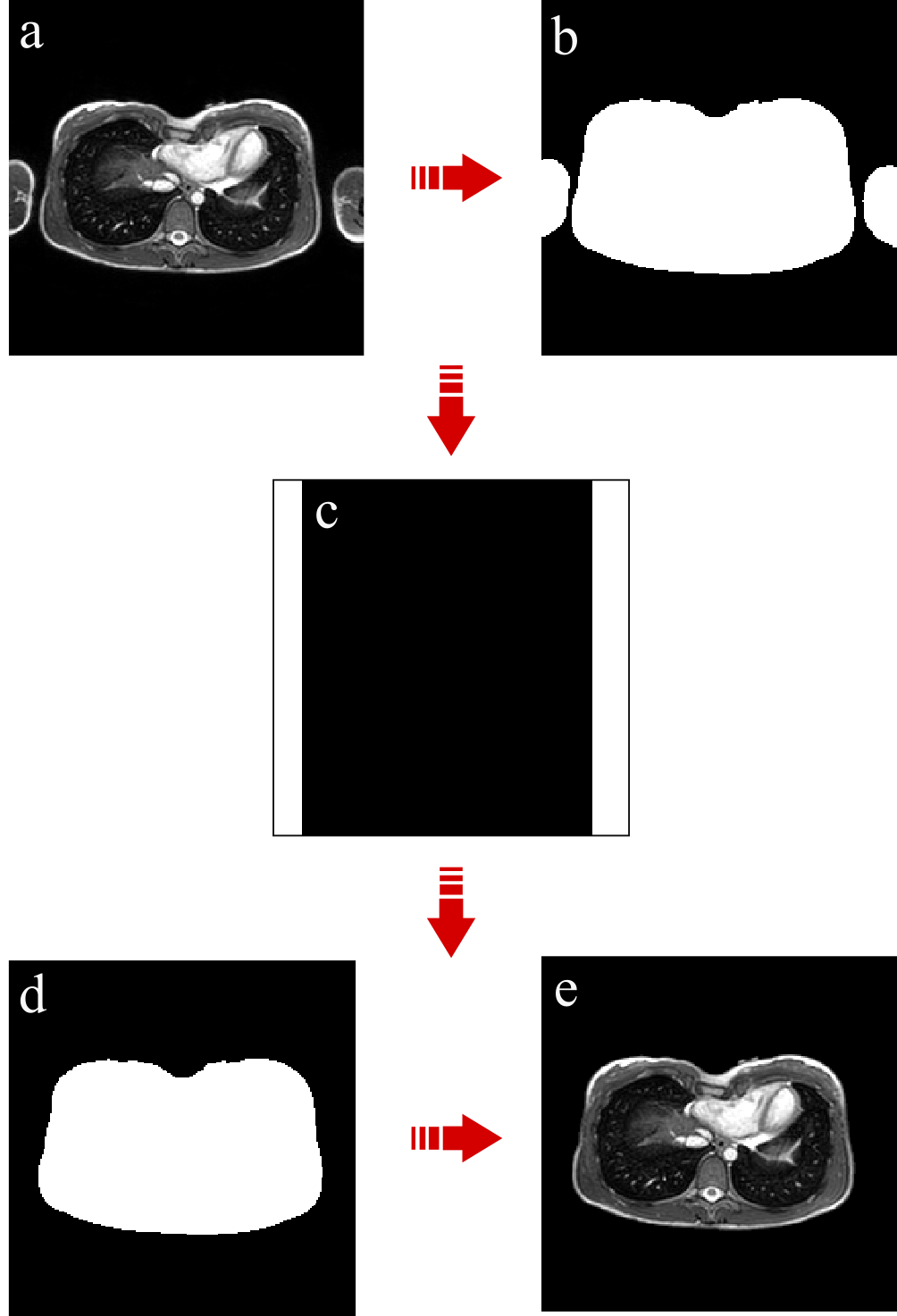


**Additional Figure 2.** **Image border correction.** Pre-processing module consists in isolating chest from other body parts if present. **a.** Due to the small dimension of chest in pediatric patients, MR images can also contain the arms or part of them on the lateral borders of the image. These are excluded through an automatic method using a properly defined mask. **b.** Firstly, the algorithm turns the grey-scale image into a binary image, by applying a manual threshold (T=0.1) to separate the foreground from the background pixels. **c.** Once the binary image is obtained, the algorithm analyzes the pixels around the lateral borders by measuring the length of the first 20 non-zero column vectors of the binary image. For each slice it finds the number of columns to delete both on the right and the left side of the image. As we noticed that area belonging to arms was nearly the same in all slices, we decided to define a unique mask, by considering the mean values, representing the number of columns on the right (c_r_) and left side (c_l_) that approximately do not belong to the chest. By doing so, we avoided potential errors in identification of the correct index, due to inhomogeneity of binary image in certain slices. In conclusion, the algorithm defines the binary mask such that all elements are assigned to 0, except the first c_r_ column and the last c_l_ columns that are assigned to. **d.** Finally, it applies the binary mask both to binary image and **e.** grey-scale image.


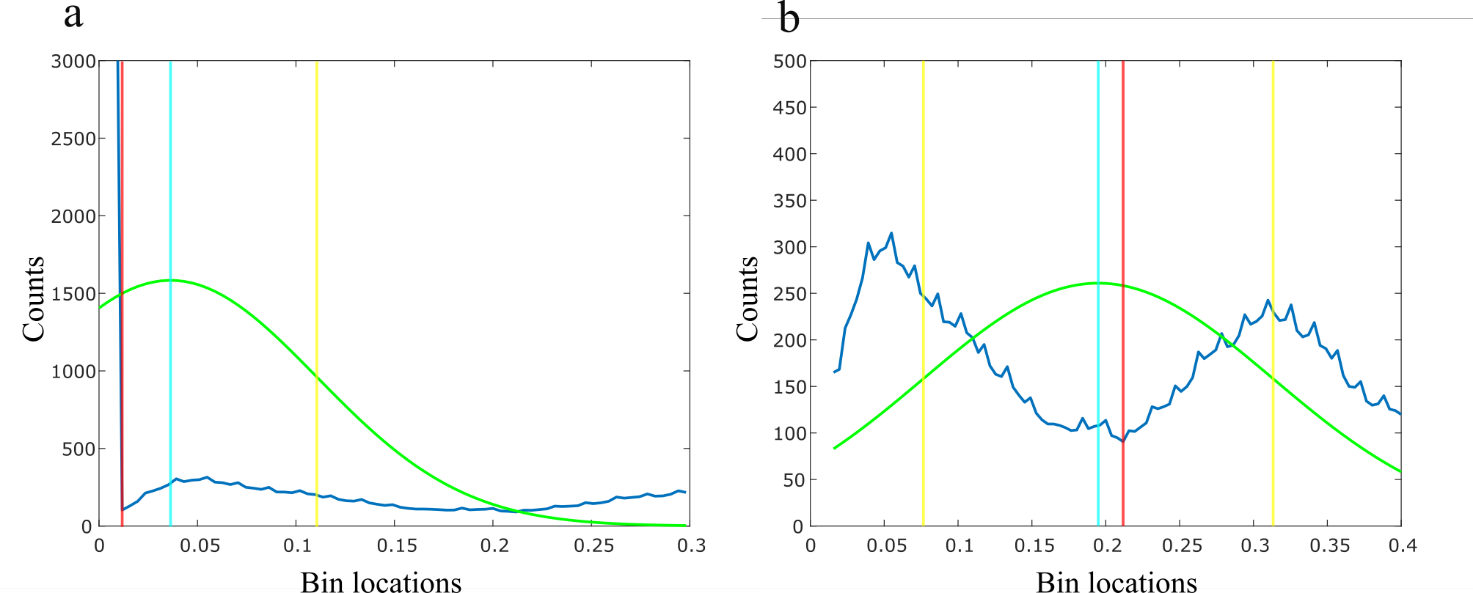


**Additional Figure 3. Histogram partitioning for lung segmentation. a.** Blue curve represents the lower part of image histogram in a continuous form (H(x)). Green curve shows the normal distribution P(x), with positions of $\mu$ and $\mu+\sigma$ identified by cyan and yellow lines, respectively. Red line represents l_bg_ threshold value found for background removal, after histogram partitioning. **b.** Blue curve represents the lower part of image histogram in continuous form, after background removal (H’(x)). Green curve shows the normal distribution P(x), with positions of $\mu$, $\mu- \sigma$ and $\mu+\sigma$, identified by cyan and two yellow lines, respectively. Red line represents l_lung_ threshold value found for lung segmentation, after histogram partitioning.


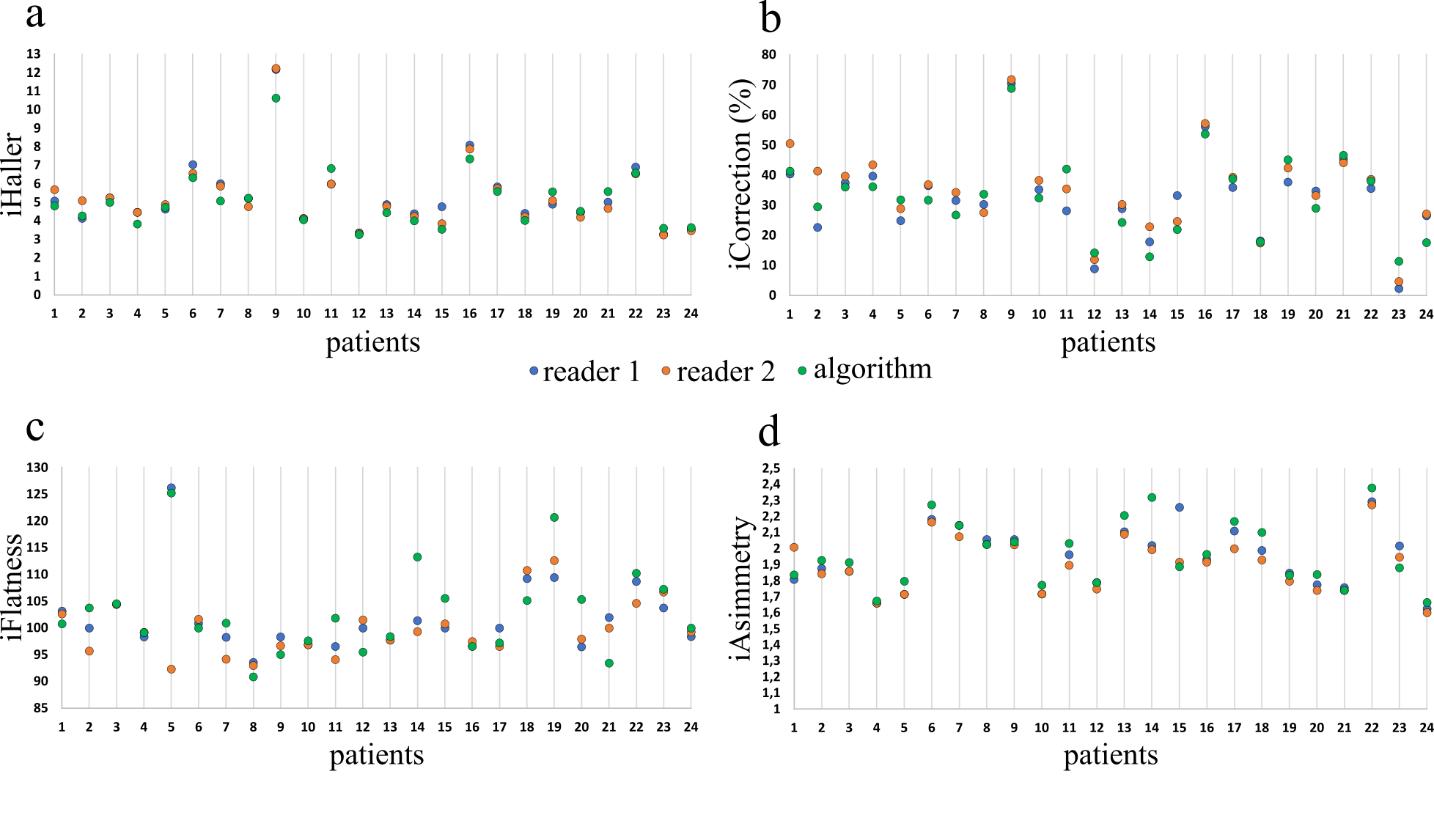


**Additional Figure 4**. **Comparison between double-blind manual measurements and automatic algorithm for computation of thoracic indexes for patients of group 1** **a.** Scatter charts representing comparison among results obtained by readers and algorithm for **a.** iHaller, **b** iCorrection, **c.** iFlatness, and **d.** iAsymmetry.
